# The correlation between skeletal muscle index of the L3 vertebral body and malnutrition in patients with advanced lung cancer

**DOI:** 10.1186/s12885-021-08876-4

**Published:** 2021-10-26

**Authors:** Xiangliang Liu, Wei Ji, Kaiwen Zheng, Jin Lu, Lingyu Li, Jiuwei Cui, Wei Li

**Affiliations:** the First Hospital of Jilin University, Xinmin St No 126, Changchun, 130021 Jilin China

**Keywords:** L3 skeletal muscle index, Advanced lung Cancer, Nutrition

## Abstract

**Background:**

Studies have shown that the skeletal muscle index at the third lumbar vertebra (L3 SMI) had reasonable specificity and sensitivity in nutritional assessment and prognostic prediction in digestive system cancers, but its performance in lung cancer needs further investigation.

**Methods:**

A retrospective study was performed on 110 patients with advanced lung cancer. The L3 SMI, the Patient-Generated Subjective Global Assessment score (PG-SGA score), body mass index (BMI), and serological indicators were analyzed. According to PG-SGA scores, patients were divided into severe malnutrition (≥9 points), mild to moderate malnutrition (≥3 points and ≤ 8 points), and no malnutrition (≤2 points) groups. Pearson correlation and logistic regression analysis were adopted to find factors related to malnutrition, and a forest plot was drawn. The receiver operating characteristic (ROC) curve was performed to compare the diagnostic values of malnutrition among factors, which were expressed by the area under curve (AUC).

**Results:**

1. The age of patients in the severe malnutrition group, the mild to moderate malnutrition group, and the no malnutrition group significantly differed, with mean ages of 63.46 ± 10.01 years, 60.42 ± 8.76 years, and 55.03 ± 10.40 years, respectively (OR = 1.062, 95%CI: 1.008 ~ 1.118, *P* = 0.024; OR = 1.100, 95%CI: 1.034 ~ 1.170, *P* = 0.002). Furthermore, the neutrophil to lymphocyte ratio (NLR) of the severe malnutrition group was significantly higher than that of the no malnutrition group, with statistical significance. The difference between the mild to moderate malnutrition group and the no malnutrition group were not statistically significant, with NLR of 4.07 ± 3.34 and 2.47 ± 0.92, respectively (OR = 1.657,95%CI: 1.036 ~ 2.649, *P* = 0.035). The L3 SMI of patients in the severe malnutrition and mild to moderate malnutrition groups were significantly lower than that of the patients in the no malnutrition group, with statistical significance. The L3 SMI of patients in the severe malnutrition group, mild to moderate malnutrition group, and no malnutrition group were 27.40 ± 4.25 cm^2^/m^2^, 38.19 ± 6.17 cm^2^/m^2^, and 47.96 ± 5.02 cm^2^/m^2^, respectively (OR = 0.600, 95%CI: 0.462 ~ 0.777, *P* < 0.001; OR = 0.431, 95%CI: 0.320 ~ 0.581, *P <* 0.001).

2. The Pearson correlation analysis showed that the PG-SGA score positively correlated with age (*r =* 0.296, *P* < 0.05) but negatively correlated with L3 SMI (*r =* − 0.857, *P <* 0.05). The L3 SMI was also negatively correlated with age (*r =* − 0.240, *P <* 0.05).

3. The multivariate analysis showed that the L3 SMI was an independent risk factor for malnutrition (OR = 0.446, 95%CI: 0.258 ~ 0.773, *P* = 0.004; OR = 0.289, 95%CI: 0.159 ~ 0.524, *P <* 0.001).

**Conclusion:**

1. The differences in the L3 SMI was statistically significant among advanced lung cancer patients with different nutritional statuses.

2. In the nutritional assessment of patients with lung cancer, the L3 SMI was consistent with the PG-SGA.

3. The L3 SMI is an independent predictor of malnutrition in patients with advanced lung cancer.

## Background

Cancer is a social and public health problem worldwide. According to statistics in 2012, the incidence and mortality of cancer in China exceeded the average level of the world [1–3]. The overall Prevalence of malnutrition in cancer is about 40%, and one fifth of mortality results from malnutrition [4]. Among all types of cancer, lung cancer occupies the third-highest Prevalence of malnutrition with 38% [5–7]. Thus, accurate and reasonable assessment of patients’ nutritional status is imperative, which can instruct nutritional support and further improve survival. Cancer-related metabolic disorder results in systemic muscle depletion due to inflammation and catabolism. However, obesity is common even in sarcopenia patients, as sarcopenia obesity, since obesity is often diagnosed with BMI rather than body composition. Therefore, accurate identification of muscle loss in patients is important. Computed tomography (CT) can accurately distinguish different components of the body and detect hidden muscle consumption, which is recognized as the gold standard for body composition analysis [8]. Martin et al. proposed skeletal muscle index at the third lumbar vertebra (L3 SMI) as a nutritional indicator that behaved well in prognosis prediction and detection of hidden muscle depletion compared to traditional nutritional indicators like BMI and weight loss, which was also demonstrated by other studies [9–14]. At present, the application value of the L3 SMI has been recognized, especially in digestive system cancers [15, 16]. But the performance of L3 SMI in lung cancer needs to be further explored. This study aimed to analyze the relationship between the L3 SMI and nutritional status of patients with advanced lung cancer and determine whether L3 SMI can be an independent predictor of malnutrition in patients with lung cancer.

## Method

### Study participants

**Fig. 1:**
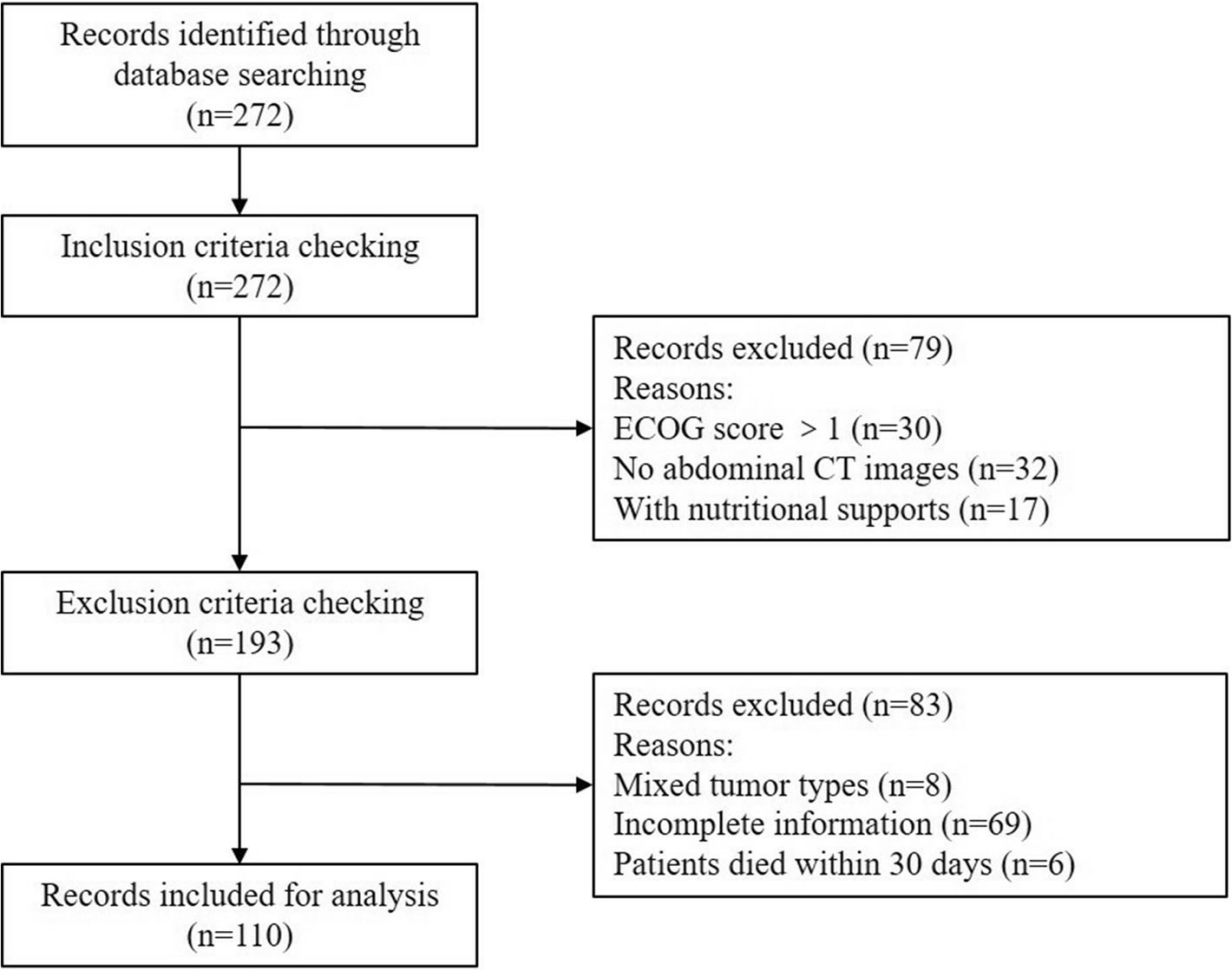
The flow diagram of patient inclusion

A retrospective analysis was performed on patients with lung cancer admitted to the Cancer Center of the First Hospital of Jilin University from January 2017 to June 2017. A total of 272 patients with lung cancer were searched from the database. Case inclusion criteria: (1) Lung cancer confirmed by pathology and TNM stage IV according to the 7th edition of American Joint Committee on Cancer;(2) The Eastern Cooperative Oncology Group (ECOG) performance status scored 0 or 1; (3) Patients with abdominal CT scan; (4) No nutritional support before assessment. Exclusion criteria: (1) Patients merged with other types of tumours; (2) Patients died within 30 days of admission; (3) Patients with incomplete materials. As shown in the study schema, 110 patients were finally included according to the inclusion and exclusion criteria (See Fig. 1).

### Data collection

All patients received PG-SGA, abdominal CT, anthropometric measurements, laboratory biochemical testing, and bioelectrical impedance analysis (BIA) within 24 h of admission. Age, sex, height, weight, and albumin (ALB), pre-albumin (PA), neutrophil-to-lymphocyte ratio (NLR) and platelet-to-lymphocyte ratio (PLR) in the peripheral blood were recorded. BMI was calculated by weight (kg)/ height ^2^ (m)^2^. BIA is a technique for measuring body composition using capacitance (Xc) and resistance (R) of biological tissues. In this study, resistance and capacitance were directly measured in ohms at 50 kHz, 800 mA by Inbody S10 (Biospace Co®). As the equation generated an indicator of body composition, phase angle (PhA) (Resistance/Capacitance) * (180/π).

SliceOmatic 5.0 software (TOMOVISION Canada) was used to analyze abdominal CT images on two consecutive planes of the L3 level. The skeletal muscle areas were measured at each plan, and the average was adopted as skeletal muscle mass, including the psoas major, erector spinae, quadratus lumbar muscles, transverse abdominis, external oblique, and internal oblique. Then the L3 SMI was calculated by skeletal muscle area (cm^2^)/height^2^(m^2^).

### Nutritional status

Nutritional status was assessed according to PG-SGA, which is composed of four worksheets on weight loss, criteria for the condition, metabolic stress and physical examination, respectively. The questionnaire has been designed to assess nutritional status in cancer patients and provides a score indicating the need for nutritional intervention. PG-SGA score ≥ 3 was defined as malnutrition.

### Statistical methods

SPSS 22.0 statistical software was used for data analysis. Continuous variables were expressed as mean ± standard deviation, and categorical variables were presented with counts (%). Patients were divided into three groups: severe malnutrition (PG-SGA ≥ 9 points, PG-SGA A group), mild-to-moderate malnutrition (3 ≤ PG-SGA ≤ 8 points, PG-SGA B group) and no malnutrition (PG-SGA ≤ 2 points, PG-SGA C group). Kolmogorov-Smirnov test was used to confirm the normal distribution of the data. Differences between groups were analyzed by T-test. Pearson correlation analysis was used for correlation analysis among variables. Univariate logistic regression was performed to analyze the association of variables and malnutrition, and all significant variables were included into multivariate logistic regression taking PG-SGA group C as the reference. Then a forest plot was drawn. The receiver operating characteristic (ROC) curve and the area under the curve (AUC) was used to explore the diagnostic value of different variables for malnutrition (PG-SGA ≥ 3 points). *P* < 0.05 indicated that the respective difference was statistically significant.

## Results

### Baseline patients information

A total of 110 patients with lung cancer were included in this study, according to the inclusion and exclusion criteria. Among them were 73 males (66.36%) and 37 females (33.64%), aged from 32 to 82 years, with an average age of 59.98 ± 9.91 years. There were 30 patients in group A, accounting for 27.3%, 54 patients in group B, accounting for 49.1% and 26 patients in group C, accounting for 23.6%. See Table 1 for details.

**Table 1:** Basic clinical information of patients

Characteristics	Patients		x^**2**^/t	P
	Inclusion (***n*** = 110)	Exclusion (***n*** = 162)		
**Sex**			0.231	0.630
**Male**	73 (66.4%)	112 (69.14%)		
**Female**	37 (33.6%)	50 (30.86%)		
**Age (y)**	59.98 ± 9.91	58.75 ± 10.42	1.231	0.677
**PA**	182.64 ± 82.77	184.77 ± 84.40	−1.721	0.354
**ALB**	38.65 ± 4.63	38.81 ± 4.45	−0.110	0.879
**BMI**	23.50 ± 3.01	23.19 ± 3.40	0.231	0.788
**PhA**	5.36 ± 0.93	5.17 ± 0.84	0.101	0.899
**NLR**	3.22 ± 2.11	2.92 ± 2.50	0.541	0.768
**PLR**	175.85 ± 104.64	169.56 ± 97.51	4.442	0.670
**L3 SMI**^**a**^	37.56 ± 9.14	36.89 ± 9.25	1.222	0.513
**Nutritional status**			2.290	0.318
**PG-SGA A**	30 (27.3%)	46 (28.40%)		
**PG-SGA B**	54 (49.1%)	66 (40.74%)		
**PG-SGA C**	26 (23.6%)	50 (30.86%)		

Nutritional status was classified according to the PG-SGA scores: PG-SGA ≥ 9 points (PG-SGA A group); 3 ≤ PG-SGA ≤ 8 points (PG-SGA B group); PG-SGA ≤ 2 points (PG-SGA C group). Continuous variables presented as mean ± s.d. and categorical variables are presented as counts (%). T test/Chi-square test was used between inclusion and exclusion patients

^a^In exclusion group, only 130 patients recorded L3 SMI

*PA* prealbumin, *ALB* albumin, *BMI* Body mass index, *PhA* phase angle, *NLR* neutrophil-to-lymphocyte ratio, *PLR* platelet-to-lymphocyte ratio, *L3 SMI* L3 skeletal muscle index

### Statistical analysis results

(1) The mean value of L3 SMI of the severe malnutrition group was 27.40 ± 4.25 cm^2^/m^2^, that of the mild-to-moderate malnutrition group was 38.19 ± 6.17 cm^2^/m^2^, and that of the no malnutrition group was 47.96 ± 5.02 cm^2^/m^2^. Statistical differences were detected in both PG-SGA A and B groups compared to the PG-SGA C group (*P* < 0.001). Besides, age, PA and PhA differed between PG-SGA A and B groups compared to PG-SGA C group (*P* < 0.005). NLR was different in severe and no malnutrition groups (*P* = 0.037), but no difference was observed between mild-to-moderate malnutrition group and severe malnutrition group (*P* = 0.097). See Table 2 for details.

**Table 2:** Basic clinical information stratified by nutritional status

***Factor***	***PG-SGA A***	***PG-SGA B***	***PG-SGA C***	t^*^	p^*^	***t***^***#***^	***p***^***#***^
	***n = 30***	***n = 54***	***n = 26***				
***Age***	63.46 ± 10.01	60.42 ± 8.76	55.03 ± 10.40	−2.422	0.018	−3.085	0.003
***PA***	145.67 ± 82.49	184.44 ± 84.80	221.53 ± 59.24	2.265	0.027	3.989	< 0.001
***ALB***	39.00 ± 2.49	39.38 ± 4.69	39.00 ± 2.49	−.0457	0.649	1.709	0.096
***BMI***	22.50 ± 3.33	24.04 ± 2.64	23.52 ± 3.14	−0.772	0.442	1.176	0.245
***PhA***	4.79 ± 0.99	5.31 ± 0.69	6.10 ± 0.78	4.572	< 0.001	5.417	< 0.001
***NLR***	4.07 ± 3.34	3.09 ± 1.47	2.47 ± 0.92	−1.683	0.097	−2.193	0.037
***PLR***	189.42 ± 118.40	182.45 ± 112.86	143.04 ± 48.72	−1.460	0.149	−1.742	0.091
***L3 SMI***	27.40 ± 4.25	38.19 ± 6.17	47.96 ± 5.02	7.022	< 0.001	16.573	< 0.001

*PA* prealbumin, *ALB* albumin, *BMI* Body mass index, *PhA* phase angle, *NLR* neutrophil-to-lymphocyte ratio, *PLR* platelet-to-lymphocyte ratio, *L3 SMI* L3 skeletal muscle index, *PG-SGA* the Patient-Generated Subjective Global Assessment

* The reference category was PG-SGA Group C, and the analysis category was PG-SGA Group B

# The reference category was PG-SGA Group C, and the analysis category was PG-SGA Group A

(2) Pearson correlation analysis showed that the PG-SGA score negatively correlated with L3 SMI (*r =* − 0.857, *P <* 0.001). As for age, ALB NLR and BMI, no relation or simply weak relation were observed among them with PG-SGA and L3 SMI. See Table 3 for details.

**Table 3:** Pearson correlation analysis

	***PG-SGA***		***L3 SMI***	
	r	p	r	p
***PG-SGA***	–	–	−0.857	< 0.001
***L3 SMI***	−0.857	< 0.001	–	–
***Age***	0.296	0.002	−0.240	0.012
***ALB***	−0.205	0.039	0.216	0.029
***NLR***	0.248	0.020	−0.314	0.003
***BMI***	−0.247	0.009	0.143	0.137

Simple Pearson correlation analysis was conducted

*PG-SGA* the Patient-Generated Subjective Global Assessment, *ALB* albumin, *NLR* neutrophil to lymphocyte ratio, *L3 SMI* the third lumbar vertebra skeletal muscle index, *BMI* body mass index

**Fig. 2:**
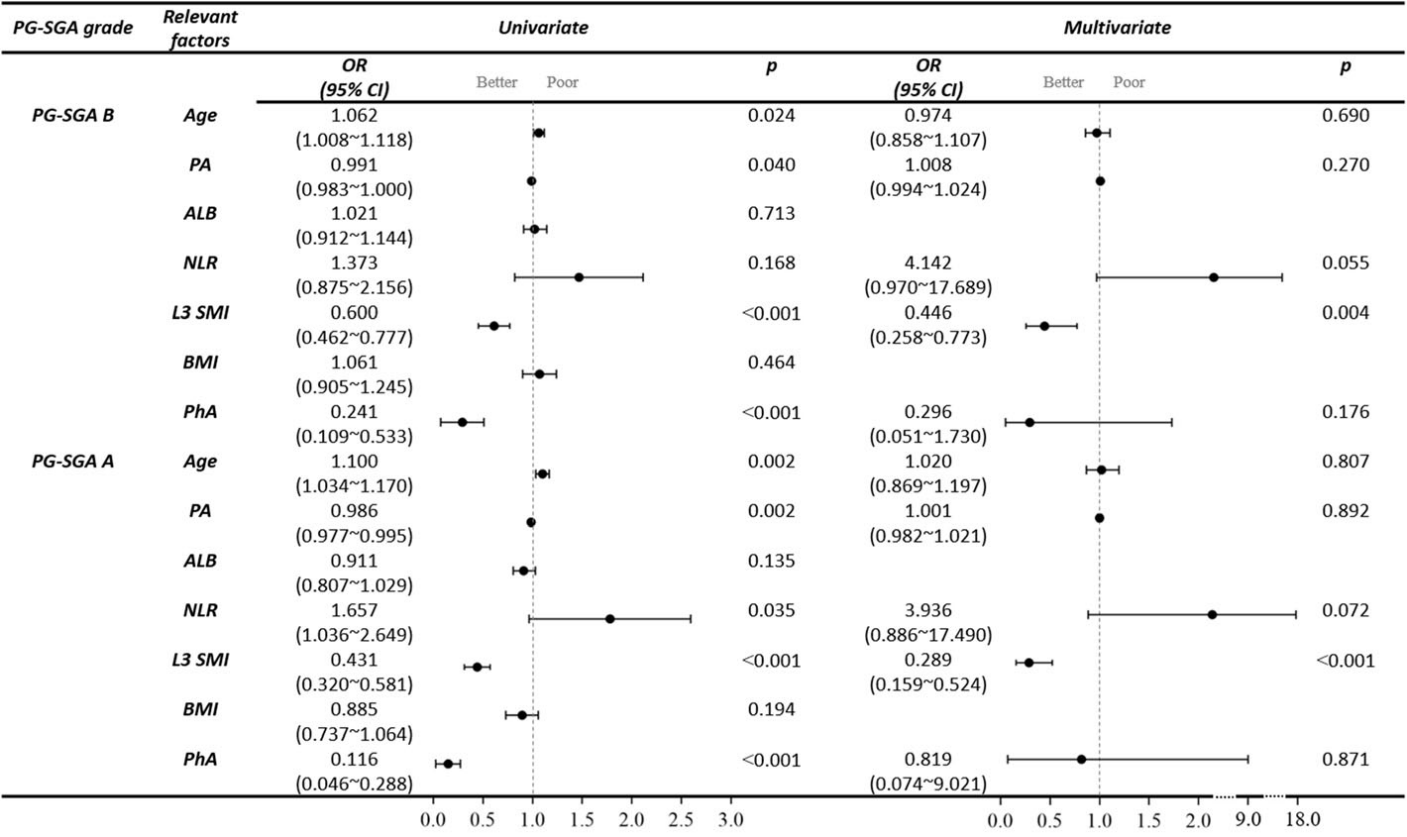
Univariate analysis and multivariate logistic regression analysis of malnutrition related factors (Attached with forest plots)

**Fig. 3:**
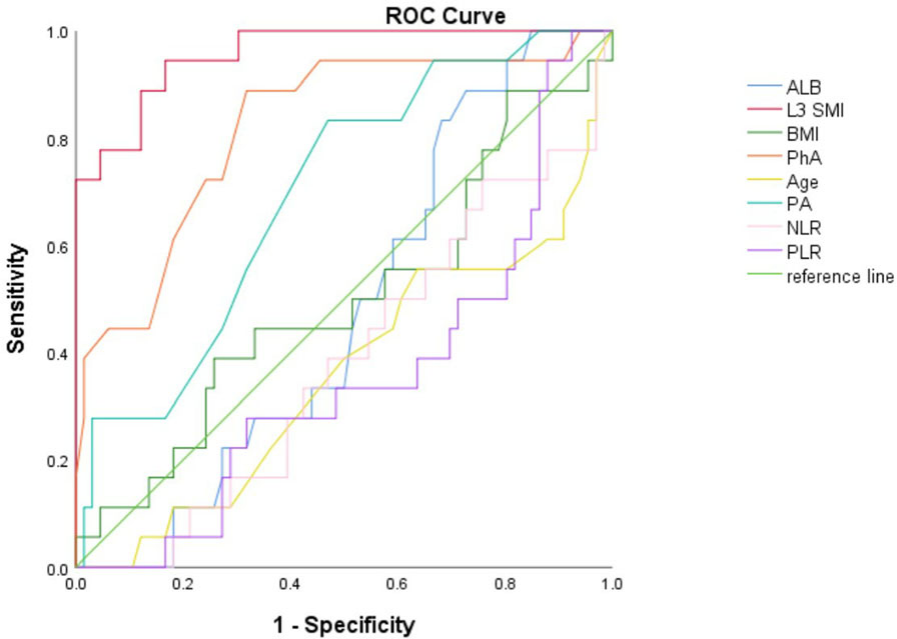
The ROC curve

(3) In patients with advanced lung cancer, the univariate analysis showed that the PA of patients in the mild-to-moderate malnutrition and severe malnutrition groups were significantly lower than that of patients in the no malnutrition group, with statistical significance (OR = 0.991, 95%CI: 0.983 ~ 1.000, *P* = 0.040; OR = 0.986, 95%CI: 0.977 ~ 0.995, *P* = 0.002). The NLR of patients in the severe malnutrition group was significantly higher than that in the no malnutrition group, with statistical significance (OR = 1.657,95%CI: 1.036 ~ 2.649, *P* = 0.035); however, there was no statistically significant difference in NLR between the mild-to-moderate malnutrition and no malnutrition groups. The PhA of patients in the mild-to-moderate and severe malnutrition groups was significantly lower than that in the no malnutrition group, with statistical significance (OR = 0.241, 95%CI: 0.109 ~ 0.533, *P* < 0.001; OR = 0.116, 95%CI: 0.046 ~ 0.288, *P <* 0.001). The L3 SMI of patients in the mild-to-moderate and severe malnutrition groups were significantly lower than that of patients in the no malnutrition group, with statistical significance (OR = 0.600, 95%CI: 0.462 ~ 0.777, *P <* 0.001; OR = 0.431, 95%CI: 0.320 ~ 0.581, *P* < 0.001). See Fig. 2.

(4) Based on the results of the univariate analysis, we included age, PA, ALB, NLR, BMI, PhA and L3 SMI as confounders in the multivariable analysis. With the no malnutrition group as the reference group, the multivariate logistic regression showed that the L3 SMI of patients in the mild-to-moderate and severe malnutrition groups statistically differed from that of patients in the no malnutrition group (OR = 0.446, 95%CI: 0.258 ~ 0.773, *P* = 0.004; OR = 0.289, 95%CI: 0.159 ~ 0.524, *P <* 0.001) and forest plots were drawn. Thus, the L3 SMI is an independent risk factor for malnutrition in advanced lung cancer. See Fig. 2 for details.

(5) Taking PG-SGA ≥ 3 points as the cut-off point for diagnosing malnutrition, the ROC curve was used to explore the diagnostic value of L3 SMI and traditional indicators. The AUC of the L3 SMI, PhA, PA, BMI, PLR and NLR were 0.958, 0.821, 0.697, 0.500, 0.357 and 0.384, respectively, which implied the novel diagnostic value of L3 SMI. See Table 4 and Fig. 3 for details.

**Table 4:** The AUC area of ROC curve

***Factors***	***AUC Area***	***95% CI of AUC Area***
***L3 SMI***	0.958	0.915 ~ 1.000
***PA***	0.697	0.569 ~ 0.826
***BMI***	0.500	0.340 ~ 0.659
***NLR***	0.384	0.242 ~ 0.525
***PLR***	0.357	0.217 ~ 0.497
***PhA***	0.821	0.706 ~ 0.935
***Age***	0.361	0.211 ~ 0.511

Taking PG-SGA ≥ 3 points as the cut-off point for diagnosing malnutrition

*PLR* platelet-to-lymphocyte ratio, *NLR* neutrophil to lymphocyte ratio, *L3 SMI* the third lumbar vertebra skeletal muscle index, *BMI* body mass index, *PhA* phase angle, *PA* prealbumin

## Discussion

Previous studies have confirmed the prognostic value of nutritional status in malignant tumors [15]. With the introduction of tumor nutrition therapy [17, 18], the importance of nutritional status of patients with tumors in clinical practice has been recognized. Researchers have also reached a consensus that accurate and comprehensive nutritional assessment, timely and effective nutritional intervention could benefit patients for a long time. Currently, the commonly used clinical nutrition assessments are roughly grouped into three categories: anthropometry indicators, laboratory inspection indicators, and comprehensive evaluation methods. Among them, the PG-SGA is the most commonly used comprehensive evaluation method. PG-SGA is a patient-involved nutritional assessment method modified on the basis of the Subjective Global Assessment (SGA). It is considered to be a specific assessment tool for the nutritional status of patients with cancer and is recommended by both the Chinese Anti-Cancer Association and the American Dietetic Association (ADA) [19, 20]. However, the PG-SGA is relatively complicated to operate, and the lack of manpower in most hospitals makes it difficult to ensure high-quality nutritional assessments. Moreover, the assessment results are easily affected by subjective factors of patients and operational deviations of medical workers, which directly lead to biased results and a lack of objective responses. The above problems have limited the clinical application of PG-SGA to a certain extent. Therefore, this study proposed the use of the L3 SMI, a quantitative, objective, accurate, and convenient indicator, to evaluate the nutritional status of patients with cancer in clinical work.

Previous studies have shown that the sensitivity and specificity of diagnosing malnutrition are the best when PG-SGA = 3 and PG-SGA = 9 are used as cut-off values [21]. The 110 patients with advanced lung cancer in this study were grouped according to PG-SGA score results. There were 30 patients in the severe malnutrition group (group A, PG-SGA ≥ 9), 54 cases in the mild-to-moderate malnutrition group (group B, 3 ≤ PG-SGA ≤ 8), and 26 cases in the no malnutrition group (group C, PG-SGA ≤ 2). The included patients were analyzed according to the groups, and patients’ age statistically differed in the mild-to-moderate and severe malnutrition groups compared with the no malnutrition group. Older patients tended to have a higher risk of developing malnutrition. Moreover, these patients were more likely to be complicated with sarcopenia, which resulted in a series of subsequent health problems. Therefore, elderly patients should pay more attention to nutritional status.

Serological examination, as one of the most widely used examinations, is an important part of the nutrition assessment of patients with cancer and has great importance in clinical work. ALB, hemoglobin, etc., are common biochemical markers that reflect nutritional status and are frequently used in clinical work. A considerable number of studies have confirmed the diagnostic value of these indicators. In recent years, new types of inflammation serological indicators, such as NLR and PLR, have gradually been recognized to represent the inflammation-immune state of patients. Relevant studies have shown that tumor inflammatory response plays an important role in malnutrition and muscle loss, which in turn coordinate and jointly promote the development of cancer [22]. The nutrition, inflammation, and immune status of patients with cancer are interrelated, and NLR has great potential for nutritional evaluation [23, 24]. In this study, we validated the role of traditional and new serological indicators in the nutritional assessment of patients with advanced lung cancer by univariate analysis. The results showed that the ALB of the malnutrition group was lower than that of the patients without malnutrition, but the difference was not statistically significant. The NLR of patients in the severe malnutrition group was significantly higher than that of the patients in the no malnutrition group.

Taking into account the invasiveness of serological examination and the complicated operation of PG-SGA, it is more likely to use a simpler and non-invasive means of nutritional assessment in clinical work, in which case, the SMI came into being. Compared with patients’ weight loss and BMI, skeletal muscle loss can more accurately and quantitatively reflect the condition of lean body tissue and nutritional status [16, 25, 26]. The core of malnutrition in patients with malignant tumors lies in the accurate identification of changes in skeletal muscle mass and strength [27]. CT examination can accurately analyze the composition of human body components. For patients with advanced tumors, without additional economic burden and radiation exposure of the patient, the CT data generated by routine examinations can be used further to evaluate the skeletal muscle content of the whole body. CT scans make it possible to accurately estimate the content of human skeletal muscle through simple operations in the clinic, and a number of studies have confirmed its accuracy and reliability [28, 29]. At the same time, CT also has the advantages of easy operation, objectiveness, and accuracy, which minimizes the resulting bias, thus having broad applications in clinical practice.

In this study, the sliceOmatic 5.0 software was used to analyze the abdominal CT of all patients and calculate the L3 SMI. The L3 SMI of patients in the severe, mild-to-moderate, and no malnutrition groups were statistically analyzed. In order to explore the application value of the L3 SMI, we conducted a Pearson correlation analysis between L3 SMI and traditional nutrition evaluation indicators. It was shown that the L3 SMI negatively correlated with the PG-SGA score with *r =* − 0.875, which indicated that the L3 SMI had a good correlation with the PG-SGA score. The L3 SMI in severe and mild-to-moderate malnutrition groups were significantly lower than those in the no malnutrition group, with statistical significance in multivariate logistic regression analysis. It could be speculated that low L3 SMI is a risk factor for malnutrition in patients with advanced lung cancer. At the same time, we also observed that ALB, NLR and age differed between the malnutrition and no malnutrition groups, and their OR values were all greater than 1. Although this difference did not reach statistical significance, it could also reflect that there were indeed changes in metabolism and inflammation in malnutrition group patients, which was consistent with the results of previous studies. We further used PG-SGA ≥ 3 points as the cut-off point for diagnosing malnutrition and drew a ROC curve to compare the L3 SMI with serological indicators. The results showed that the L3 SMI had a good diagnostic efficacy on malnutrition in patients with advanced lung cancer, and the diagnostic efficacy was even better than the serological index. These results suggested that the L3 SMI is consistent with the serological index and PG-SGA, which largely confirmed its application value in the nutritional assessment of patients with advanced lung cancer.

The decrease in SMI was obvious in advanced lung cancer patients who developed malnutrition. We believe that this is because changes in skeletal muscle mass and function occur over a long-term accumulative process, which is affected by the side effects of anti-tumor therapy, tumor biological characteristics, nutrition consumption, abnormal energy metabolism, and other factors. Malnutrition in patients with malignant tumors is affected by tumor type, stage, location, treatment method, and others [30, 31]. Compared to patients with digestive system neoplasms, whose malnutrition are often caused by tumor space effects and digestive tract dysfunction, malnutrition in patients with advanced lung cancer is often caused by a combination of systemic factors [5, 32, 33]. Long-term anti-tumor treatment and chronic consumption from tumors can lead to changes in body composition. The reduction of skeletal muscle and lean body tissue directly leads to treatment-related adverse reactions and worse treatment efficacy. On the other hand, continuous skeletal muscle consumption decreases the patients’ tolerance to treatment and increases the risk of malnutrition. Malignant tumors can also secrete pro-inflammatory factors that increase fat and protein catabolism leading to weight loss. This reflects the interaction of inflammation, immunity, and nutrition in patients with advanced lung cancer [34–36]. Long-term changes in the above factors eventually lead to significant skeletal muscle reduction and weakening of strength in patients with malnutrition.

To our knowledge, this study is the first to apply the L3 SMI to nutritional assessment in patients with advanced lung cancer. The results of this study provide a strong verification for promoting the application of L3 SMI in nutritional status assessments of patients with cancer. The L3 SMI reflects the state of the patient’s skeletal muscle. The decrease in skeletal muscle mass is related to patients’ immune function and inflammation state [37, 38], which means that, to a certain extent, the SMI reflects the immune-inflammatory state of the body and represents changes in body nutritional metabolism and composition. In summary, the L3 SMI provides a quantitative index for the nutritional assessment of patients with advanced lung cancer. Nonetheless, there were deficiencies in this study, and further exploration is needed. First, a larger sample size for further verification is needed. Second, in terms of the research objects, we look forward to a more detailed grouping design that construct L3 SMI group cut-off values, which are suitable for different populations according to sex or cancer type.

In summary, a low L3 SMI is an independent risk factor for malnutrition in patients with advanced lung cancer. We believe that the L3 SMI has excellent sensitivity and specificity for the nutritional assessment of patients with advanced lung cancer, which is consistent with the PG-SGA and has good efficacy in diagnosing malnutrition. The L3 SMI obtained by CT, as a new nutritional evaluation index, has the advantages of being non-invasive, objective, and accurate, thus being a promising quantitative evaluation index in the future.

## Conclusion

To sum up, the correlation between the L3 SMI and malnutrition was investigated. We chose the Asian population as the research object, first observed that the L3 SMI statistically differed among patients with advanced lung cancer with different nutritional status. The regression model suggested the L3 SMI was an independent risk factor for malnutrition, and we further confirmed the applicability of it in advanced lung cancer patients. The L3 SMI has good diagnostic value in nutritional assessment.

## Data Availability

Materials described in the manuscript, including all relevant raw data, will be freely available to any scientist wishing to use them for non-commercial purposes, without breaching participant confidentiality. Any investigator interested in viewing raw data may contact us by email: ds9291@qq.com.

## Abbreviations

SMI: Skeletal muscle index
the L3: The third lumbar vertebra
BMI: Body mass index
ALB: Albumin
PA: Prealbumin
NLR: Neutrophil to lymphocyte ratio
PLR: Platelet to lymphocyte ratio
PG-SGA: The Patient-Generated Subjective Global Assessment
SGA: The Subjective Global Assessment
BIA: Bioelectrical impedance analysis
ROC: The Receiver Operating Characteristic Curve
PhA: Phase angle
ADA: The American Dietetic Association
